# Breast Cancer: From Pathophysiology to Novel Therapeutic Approaches 2.0

**DOI:** 10.3390/ijms24032542

**Published:** 2023-01-29

**Authors:** Antonella Zannetti

**Affiliations:** Institute of Biostructures and Bioimaging, National Research Council (IBB-CNR), 80145 Naples, Italy; antonella.zannetti@cnr.it

Breast cancer (BC) is the most common malignancy in women worldwide. It is a very heterogeneous disease characterized by different molecular profiles that are clinically divided into three main subtypes by hormone receptor (ER and PR) and HER2 (ERBB2) status: luminal ER-positive and PR-positive, which is further subdivided into luminal A and B, HER2-positive, and triple-negative breast cancer (TNBC) [1]. To date, in the fight against breast cancer, the screening program is the most important aid in reducing mortality caused by this disease. Conventional chemo- and radiotherapy, often including targeted agents, represent the main systemic treatment used for BC patients, although many develop drug resistance, relapse, and secondary metastases [1]. The early stage of BC is considered curable in 70–80% of patients due to improved multimodal therapy, while advanced metastatic BC is an unmet challenge. Many studies are ongoing, and efforts are being made to identify new molecular biomarkers to improve diagnostic and therapeutic approaches. Therefore, the aim of this Special Issue is to highlight the current preclinical and clinical studies conducted in breast cancer research to shed light on the deep molecular signatures and the next therapeutic scenario of this highly heterogeneous disease.

All of the articles published in “Breast Cancer: From Pathophysiology to Novel Therapeutic Approaches 2.0” meet these criteria and report interesting results, often using transcriptomic approaches, on the identification and implication of novel biomarkers that are able to discriminate between different types of BC and between responder and non-responder patients. In addition, new possible prognostic factors related to overall survival have been investigated, but most importantly, new targeted and less toxic therapies have been evaluated for use alone or in combination with chemotherapeutic agents.

In an interesting review, Chiodo et colleagues [2] clarified the contribution of steroid hormones and their receptors in the growth, development, and lifetime changes of the mammary gland as well as its crucial role in breast cancer development and progression. They elucidated the controversial impact of androgens/AR in preventing or promoting breast cancer and the signaling pathways involved. 

It is noteworthy that by analyzing the TCGA/GTEx datasets available within GEPIA2 and using the tissue microarray of a cohort of 252 samples, a study showed that breast cancer patients with higher methionine adenosyltransferases 2 (MAT2A) and no MAT1A had a worse survival rate (*p* = 0.0057). The authors found that a higher cytoplasmic/nuclear (C/N) MAT2A protein expression ratio correlated with poor overall survival, and a MAT2A C/N expression ratio ≥ 1.0 was determined as an independent risk factor by Cox regression analysis [3]. 

del Mar Noblejas-López et al. [4] demonstrated, using the transcriptomic mapping of cyclin-dependent kinases (CDKs), that the expression of CDK9 predicted a detrimental outcome in basal-like breast cancer and, particularly, in the luminal B subtype with HER2+ expression. They found that a novel proteolysis targeting chimera (PROTAC) compound against CDK9, THAL-SNS-032, showed a major anti-tumoral effect on breast cancer cell lines, expressing both ER and HER2, such as BT474. Interestingly, drug-resistant cells derived from BT474 cell line with resistance to trastuzumab (BT474-RH), to the antibody drug conjugate TDM1 (BT474-TDM1R), or to the kinase inhibitor lapatinib (BT474-LAPA-R) displayed a particular sensitivity to THALSNS-032. Conversely, low doses of the compound induced severe toxicity in BT474 xenografts, in particular in in the gastrointestinal epithelium, without causing a significant reduction of tumor volume, thus showing its inverse therapeutic index. 

A synergic antitumoral effect of Ruxolitinib (an oral selective inhibitor of Janus kinases 1 and 2) and Calcitriol in HER2-enriched and triple negative subtypes of breast cancer cells was reported by Schneider and colleagues [5]. The combined treatment caused cell proliferation inhibition, apoptosis induction, cell cycle arrest, and the alteration of the cell signaling protein expression underlying these mechanisms. Similar results were obtained in vivo with Ruxolitinib and Calcitriol that showed a synergistic inhibitory effect on tumor growth in MDA-MB-468 xenografts, corroborating the in vitro observations on the potential of this treatment in certain subtypes of breast cancer. 

To improve the selectivity and bioavailability of chemotherapeutic agents, such as the platinum complexes class and overcome multiple mechanisms of drug resistance, Czarnomysy et al. [6] synthesized a novel imidazole platinum(II) complex conjugated with the second-generation polyamidoamine (PAMAM) dendrimer (PtMet2–PAMAM). They showed that this compound increased apoptosis via caspase-9 (intrinsic pathway) and caspase-8 (extrinsic pathway) as well as triggered autophagy through p38 pathway activation in different breast cancer cells. Furthermore, the complex inhibited drug efflux transporters and its positive charge increased the tumor cell uptake, demonstrating the ability of PtMet2–PAMAM to reverse multidrug resistance [6]. 

TNBC represents one of the most aggressive types of BC because of its high heterogeneity and plasticity [7]. Currently, chemotherapy still remains the main therapeutic option, including taxane- and anthracycline-based treatments for TNBC, despite the high rate of non-responders due to the occurrence of drug resistance. Interestingly, a study by Gangapuram et al. [8] compared the transcriptome profile of TNBC cells in response a novel cytostatic compound, a tetrahydroisoquinoline named GM-4-53, and paclitaxel. Both treatments caused, in TNBC cells, changes in transcripts that influence microtubule spindle formation, chromosome segregation, mitosis/cell cycle, and transforming growth factor (TGF) signaling as well as the downregulation of “inhibitor of DNA Binding/Inhibitor of differentiation” (ID) transcripts, amphiregulin (AREG) and epiregulin (EREG), with GM-4-53 having more significant effects than paclitaxel. The authors hypothesize that given the efficient solubility of GM-4-53, its low molecular weight (MW; 296) and its ability to penetrate a small solid tumor mass and effectively block the cell cycle, this drug may have future therapeutic value in treating TNBC. The effect of desethylamiodarone (DEA), the major metabolite of amiodarone (AM), as anti-tumoral agent was investigated by Gallyas and colleagues [9] in TNBC cells. They found that DEA reduced tumor cell viability, proliferation, and invasion; lowered mitochondrial transmembrane potential; and induced mitochondrial fragmentation. In addition, DEA-resistant TNBC cells showed an upregulation of cyclooxygenase 2 (COX-2) and its activity was counteracted by the specific inhibitor celecoxib. 

Several innovative approaches are being explored to overcome limitations due to the lack of well-defined TNBC biomarkers. Recently, Camorani et al., using a TNBC cell-SELEX approach, generated a panel of six RNA aptamers able to bind at high efficiency to the surface proteins of human TNBC cells without recognizing non-malignant cells or non-TNBC breast cancer cells [10]. Aptamers recognize their targets with high affinity and specificity in a manner similar to antibody–antigen interactions and have been used to specifically deliver through nanovector-based chemotherapeutic agents and immune checkpoint inhibitors in TNBC cells [11,12]. In a study published in this Special Issue, Camorani et colleagues [13] optimized three of six aptamers by shortening and proved that the truncated aptamers showed superb nuclease resistance, specific binding to TNBC target cells, and rapid internalization into acidic compartments and also hampered the growth of TNBC cells as mammospheres.

In conclusion, taken together, the studies published in this Special Issue demonstrate that the future of research in BC, through the deeper knowledge of molecular mechanisms associated with drug resistance and underlying tumor progression, is reaching the point where treatments can be tailored to each individual patient.
